# Validation of the MINI (DSM IV) Tool for the Assessment of Alcohol Dependence among Young People in Northern Tanzania Using the Alcohol Biomarker Phosphatidylethanol (PEth)

**DOI:** 10.3390/ijerph121114021

**Published:** 2015-10-30

**Authors:** Joel M. Francis, Anders Helander, Saidi H. Kapiga, Helen A. Weiss, Heiner Grosskurth

**Affiliations:** 1 Department of Infectious Disease Epidemiology, London School of Hygiene and Tropical Medicine, Keppel Street, London WC1E 7HT, UK; E-Mails: Saidi.Kapiga@lshtm.ac.uk (S.H.K.); Helen.Weiss@lshtm.ac.uk (H.A.W.); Heiner.Grosskurth@lshtm.ac.uk (H.G.); 2 National Institute for Medical Research, Mwanza Centre, Mwanza, Tanzania; 3 Department of Laboratory Medicine, Karolinska Institutet, Stockholm SE-141 86, Sweden; E-Mail: anders.helander@ki.se; 4 Mwanza Intervention Trials Unit (MITU), Mwanza, Tanzania

**Keywords:** MINI, DSM IV, AUDIT, PEth, alcohol dependence, young people, Tanzania

## Abstract

The alcohol dependence section of the Mini International Neuropsychiatric Interview questionnaire (MINI) has not been evaluated in young Africans. We applied the MINI in a cross-sectional study of 202 alcohol users from northern-Tanzania, aged 18–24 years (103 male casual workers and 99 students), and validated it against phophatidylethanol (PEth) at a cut-off suggesting heavy chronic alcohol use (≥0.30 µmol/L). Blood was assayed for PEth (16:0/18:1-subform) by liquid chromatography-tandem mass spectrometry. The MINI dependence criteria (≥3 positive responses) were met by 39% participants although their PEth levels were low. Contrary, many young people with high PEth levels were not classified as dependent. The sensitivity of the MINI ranged from 0% to 69% (female students and male workers, respectively) and specificity from 52% to 85% (workers and female students, respectively). The highest AUROC (0.68) occurred with a cut-off of ≥4 positive responses. A modified MINI with three affirmative responses to five questions increased specificity to 92%–97%; however, sensitivity remained low. The performance of the MINI in detecting dependence among young people from northern-Tanzania is unsatisfactory. Specificity was improved using a modified version but sensitivity remained low. An accurate tool for the diagnosis of alcohol dependence is needed for epidemiological and clinical purposes.

## 1. Introduction

Harmful alcohol use in young people is becoming an increasingly significant public health problem in low-income settings, including sub Saharan Africa (SSA) [1]. The problem is also common among young people in East Africa but, due to lack of validated diagnostic tools, the diagnosis of harmful use and alcohol dependence among young people remains challenging [2].

The Diagnostic and Statistical Manual of Mental Health Disorders, 4th edition (DSM IV) and the International Classification of Diseases, version 10 (ICD-10) are acknowledged tools for the diagnosis and classification of mental health disorders, including alcohol use disorders [3,4]. These instruments are utilized both in low- and high-income settings. The Mini International Neuropsychiatric Interview questionnaire (MINI) is based on the DSM IV/ICD 10 and is widely used to identify patients with suspected alcohol abuse and alcohol dependence in clinical and research settings [5] (see also Appendix 1). DSM IV has recently been replaced by DSM-5. In DSM-5, the formerly distinct diagnoses of substance abuse and dependence have been unified into one diagnosis (“substance use disorder”; SUD) for which the originally separate lists of diagnostic criteria have been merged and slightly modified [6,7]. However, the MINI continues to be used in research on alcohol related disorders [8].

The application of the DSM IV/DSM-5 and MINI diagnostic criteria in young people is challenging and has been a subject of debate for many years, due to two highly sensitive questions on craving for and compulsion to alcohol use that may be non-specific when applied to those who started using alcohol recently [9,10,11]. These questions are “*Did you need to drink a lot more in order to get the same effect you got when you started first drinking or did you get much less effect with continued use of the same amount?*” (MINI question I2a), and “*During the times when you drank alcohol, did you end up drinking more than you planned when started?*” (MINI question I2c). The questions do not sufficiently take developmental issues and young people’s perceptions and intentions into account, and responses may therefore be misleading.

This lack of specificity may also affect the application of the MINI in the context of alcohol use among young people in SSA. In our study, we aimed to explore the validity of the MINI version 6.0 in detecting alcohol dependence, by applying it to young drinkers recruited from a large city in East Africa. Because there is no objective (*i.e.*, non-self-report based) gold-standard methodology to diagnose dependence, we used an assay to detect phosphatidylethanol (PEth), a specific and sensitive blood biological marker indicating current chronic alcohol use, to validate the MINI [12,13,14]. We did this based on the assumption that alcohol dependence among young people will usually be associated with continued or episodic excessive alcohol intake [15]. PEth is an ethanol metabolite found in blood that has a comparatively long detection time, and the assay can be applied to identify excessive alcohol use that occurred over recent weeks to ~1 month, and to discriminate roughly between levels of alcohol use. PEth has been previously used to compare self-reported hazardous and harmful alcohol use in SSA [14,16,17,18,19,20]. For this paper, we have utilized the harmonized PEth cut-off (≥30 µmol/L of the major subform PEth-16:0/18:1) that is the standard level used in the Swedish population to indicate heavy alcohol use [21]. To our knowledge, this is the first study using an alcohol biomarker to validate the MINI for alcohol dependence among young people in Africa.

## 2. Experimental Section

### 2.1. Study Population and Procedures

In March and April 2014, we conducted a cross-sectional study among two groups of young people (college students and casual labourers) in Mwanza city, northern Tanzania. Male and female college students were recruited from higher learning institutions, and casual labourers comprised young men working in garages (car workshops). Casual workers from this sector are typical for male workers with unstable employment in this geographical setting, and can be more easily identified than for example casual workers from temporary building sites. We recruited young people aged 18–24 years who reported to have consumed alcohol at least once in the last year. Earlier research in this population showed that alcohol use is initiated early, with a median age of 17 years [22]. We enrolled those who provided written informed consent, and impartial witnesses documented the consent forms for illiterate study participants. None of the study participants reported to be under the influence of alcohol at the time of the interview. Ethical approval was received from the Lake Zone Institutional Review Board at the National Institute for Medical Research (NIMR) Mwanza centre (MR 53/100/155) and the London School of Hygiene and Tropical Medicine (LSHTM ethics ref 7074).

We randomly selected one class each at two randomly chosen colleges out of five colleges, and enrolled all eligible and consenting students. We consecutively visited garages in Mwanza city starting with large garages and enrolled all eligible casual workers until we attained the desired sample size. The study was performed by two young trained research assistants who administered the Alcohol Use Disorders Identification Test (AUDIT) [23] and two medical officers who administered the MINI and drew blood samples after having been trained in the application of this tool for one week by a specialist psychiatrist jointly with the principal investigator. Study participants and research assistants were matched by gender for comfortability during the interviews and to minimize bias.

### 2.2. Sample Size

We chose a sample of 200 young alcohol users with the aim of achieving good precision for the detection of alcohol dependence by MINI against PEth at a cut-off level that is routinely employed to indicate recent chronic heavy use (PEth-16:0/18:1 ≥ 0.30 µmol/L) [21]. Assuming a true prevalence of alcohol use disorders including possible dependence among young people in eastern Africa of 15% [2], a sample of 200 young people would provide reasonable precision for estimates of sensitivity and specificity. For example, for a sensitivity of 80% or 95% we would obtain 95% confidence intervals (CI) of 61.4%–92.3% and 77.9%–99.1%, respectively. For a specificity of 80% or 95%, the 95% CI would be 73.2%–85.7% and 90.9%–98.0%, respectively.

### 2.3. Measurement of Self-Reported Alcohol Use and Alcohol Dependence

Self-reported alcohol use was documented using the AUDIT and the alcohol dependence related Section I2 of the MINI version 6.0 [5]. This section of the MINI contains seven questions derived from DSM IV that are designed to detect possible alcohol dependence (Appendix 1).

### 2.4. Blood Sample Collection, Processing and Laboratory Assay for Phosphatidylethanol (PEth)

Each study participant was asked to provide 5 mL of venous blood collected into EDTA vacutainer tubes. Before collection, the venipuncture site was swabbed twice with clean water and allowed to dry. Field workers were instructed not to use alcohol for sterilisation of the skin. The blood samples were immediately stored in a cool box in the field, and transferred to the NIMR laboratory in Mwanza within 3 h where they were kept at −80 °C [24]. Samples were then sent on dry ice to the Karolinska Institutet and Karolinska University Laboratory (Stockholm, Sweden) for assay of PEth-16:0/18:1, the main PEth homologue in human blood [13], using liquid chromatography-tandem mass spectrometry (LC-MS/MS). In the laboratory, samples were stored at −80 °C until taken for LC-MS/MS analysis, using selected ion monitoring (SIM) in negative mode of the deprotonated molecules [25]. The detection limit for whole blood PEth-16:0/18:1 is 0.01 µmol/L. The routinely applied cut-off used to indicate any intake of alcohol is ≥0.05 µmol/L, and ≥0.30 µmol/L to indicate heavy drinking; the higher cut-off was used to indicate alcohol dependence for this study [16,21,26].

### 2.5. Data Management and Analysis

Data were double-entered into computers at the data management unit of the Mwanza Intervention Trials Unit (MITU) at NIMR, using version 3 of the Open Clinica software (OpenClinica, LLC). PEth concentration data were merged with the questionnaire data, and data were exported to Stata 13 for analysis (StataCorp, College Station, TX, USA).

The MINI criteria indicating dependence are met when ≥3 of the seven questions in Section I2 are answered with “Yes” (Appendix 1). We determined the sensitivity and specificity of the resulting classification against PEth at a level of ≥0.30 µmol/L. We also generated a modified version of the MINI in which the first and third items (MINI questions I2a and I2c) were excluded due to their lack of specificity, and defined dependence as meeting three out of the five remaining questions. We also validated both versions of the MINI for other cut-offs in the number of positively answered questions. For each possible cut-off level, we calculated the area under the receiver operating characteristic (AUROC).

Primary outcomes were: (i) the sensitivity and specificity of the original MINI criteria, and (ii) the sensitivity and specificity of varied cut-off points of the modified MINI, both calculated against a diagnosis of heavy alcohol use as determined by PEth (>0.30 µmol/L) [25].

In addition, for different MINI response rates we computed respondents’ total AUDIT scores and corresponding median and interquartile ranges. The standard AUDIT cut-off score of ≥8 was used to categorise “hazardous/harmful alcohol use or possible alcohol dependence” [27].

## 3. Results

### 3.1. Characteristics of the Population

We recruited 202 young people that comprised 103 (51%) male casual labourers, 58 (29%) male college students and 41 (20%) female college students. Women were not employed in any of the garages from where casual workers were recruited. The majority (166; 82%) of the participants were aged above 20 years. Approximately half (107; 53%) had an AUDIT score of ≥8, with male casual labourers reporting this most frequently (66%). The median AUDIT score was highest among male casual labourers (10; IQR: 6–16) and lowest among female college students (5; IQR: 3–8). Nine (9%) of male casual labourers were suspected to be alcohol dependent by AUDIT criteria, and fewer than 4% of college students. Twenty five (12.4%) participants had PEth concentration of ≥0.30 µmol/L. Specifically, two (4.9%) female college students, seven (12.1%) male college students and 16 (15.5%) male casual labourers had PEth concentrations of ≥0.30 µmol/L (Table 1).

**Table 1:** General characteristics of the study population.

Characteristic	Categories	Overall	Female College Students	Male College Students	Casual Labourers
**Sample (N)**		202	41	58	103
**Age (%)**	18–20 years	36 (17.8)	4 (9.8)	2 (3.5)	30 (29.1)
	21–24 years	166 (82.2)	37 (90.2)	56 (96.6)	73 (70.9)
**Religion (%)**	Moslem	36 (17.8)	8 (19.5)	5 (8.6)	23 (22.3)
	Catholic	102 (50.5)	20 (48.8)	32 (55.2)	50 (48.5)
	Other Christians	64 (31.7)	13 (31.7)	21 (36.2)	30 (29.1)
**Education (%)**	Primary and less	62(30.7)	0(0.0)	0(0.0)	62(60.2)
	Secondary and above	140 (69.3)	41 (100.0)	58 (100.0)	41 (39.8)
**Marital status (%)**	Single	64 (31.8)	9 (22.0)	19 (33.3)	36 (35.0)
	In relationship	137 (68.2)	32 (78.1)	38 (66.7)	67 (65.0)
**Age at alcohol initiation (%)**	Less than 18 years	116 (58.0)	22 (53.7)	34 (59.7)	60 (58.8)
	18–24 years	84 (42.0)	19 (46.3)	23 (40.4)	42 (41.2)
**AUDIT( 10 items) score** **Median(IQR)**		8.5 (5.0,14.0)	5.0 (3.0–8.0)	7.0 (5.0, 13.0)	10.0 (6.0, 16.0)
**AUDIT (%)**	<8 (Low risk drinking)	95 (47.0)	30 (73.2)	30 (51.7)	35 (34.0)
	≥8 (Risk drinking)	107 (53.0)	11 (26.8)	28 (48.3)	68 (66.0)
**Dependence by AUDIT dependence questions (%)**	No	190 (94.1)	40 (97.6)	56 (96.6)	94 (91.3)
	Yes	12 (5.9)	1 (2.4)	2 (3.5)	9 (8.7)
**PEth cut-off for heavy alcohol intake (PEth-16:0/18:1 > 0.30 µmol/L)** **(%)**	Yes	25 (12.4)	2 (4.9)	7 (12.1)	16 (15.5)

### 3.2. Responses to MINI Questions and Characteristics of Respondents

Most participants 177 (88%) responded with “Yes” to MINI question I2a “*Did you need to drink a lot more in order to get the same effect you got when you started first drinking or did you get much less effect with continued use of the same amount?*” (Table 2). The median AUDIT score among these participants was 9 (IQR: 6–15) and was only 5 (IQR: 2–6) among those who responded with “No”. MINI question I2c “*During the times when you drank alcohol, did you end up drinking more than you planned when started?* “was answered affirmatively by 121 (63%) participants, among whom the median AUDIT score was 11 (IQR: 7–17) whilst it was 5 (IQR: 3–9) among those responding with “No”. For the remaining questions, the proportion with a positive response ranged between 7%–28% with median AUDIT scores ranging from 12–16 (Table 2). Overall, 79/202 (39%) participants responded affirmatively to three or more MINI I2 questions and thus met the criteria for dependence; 15%, 35% and 51% among female students, male students and male casual workers, respectively, (Table 3). Because of these findings, we regarded questions MINI I2a and I2c as too non-specific to detect alcohol dependence and excluded them from the modified version of MINI.

### 3.3. Sensitivities and Specificities of the Original and Modified Versions of MINI Compared to PEth.

The sensitivity of the original version of the MINI when compared to the PEth cut-off for heavy drinking (≥0.30 µmol/L) was low, ranging from 0% (among female college students) to 69% (among male casual labourers) (Table 3). The specificity ranged from 52% (male causal labourers) to 85% (female college students). A positive response to ≥5 questions yielded an overall sensitivity of 12% and specificity of 94%. Using the modified version of the MINI (with ≥3 “Yes” responses out of five selected DSM IV questions), specificity against PEth (≥0.30 µmol/L) was high in all groups (92%–97%), however sensitivity was poor (ranging from 0–14% across groups); *i.e.*, few of those with positive PEth results scored positive on the modified MINI questionnaire (Table 4). The highest AUROC (0.68) was achieved when applying the original MINI with a cut-off level of ≥4 positive responses, in the group of male college students (Table 3).

## 4. Discussion

The MINI when applied with the original criteria (≥3 “Yes” responses to seven dependence questions) showed low sensitivity and moderate specificity among young people in northern Tanzania when using the specific and sensitive alcohol biomarker PEth with a cut-off for heavy drinking (≥0.30 µmol/L PEth-16:0/18:1) as a proxy for objectively assessing alcohol dependence. Using a higher PEth cut-off than 0.30 µmol/L (such as ≥0.40 µmol/L) did not improve sensitivity or specificity. The sensitivity was low because many participants with confirmed heavy chronic alcohol use did not respond positively to the relevant MINI screening questions. The lack of specificity resulted mainly from the two questions related to alcohol tolerance and compulsion to drink that were answered positively by most young people in our study. This problem has been observed by others and the two questions have been subject of debate for many years [9,10]. Our modified version of the MINI (excluding questions I2a and I2c and using a cut-off point of ≥3 “Yes” response) was highly specific but still had low sensitivity. The same applied if the original 7-question MINI tool was used with a higher cut-off of ≥5 positive responses.

**Table 2:** Responses to MINI DSM IV questions compared to AUDIT scores and PEth test results.

Variables	Responses	*N* (%)	Dependent by Original DSM IV Criteria *N* (%)	PEth-16:0/18:1 ≥0.30 µmol/L *N* (%)	Median(IQR) AUDIT Score
**DSM IV—Dependence question 1:Did you need to drink a lot more in order to get the same effect you got when you started first drinking or did you get much less effect with continued use of the same amount?**	Yes	177 (87.6)	79 (44.6)	25 (14.1)	9 (6,15)
	No	25 (12.4)	0 (0.0)	0 (0.0)	5 (2,6)
**DSM IV—Dependence question 2: When you cut down on drinking did your hands shake, did you sweat or feel agitated? Did you drink to avoid these symptoms (for example, “the shakes”, sweating or agitation) or to avoid being hungover?**	Yes	17 (8.4)	17 (100.0)	5 (29.4)	13 (7,19)
	No	185 (91.6)	62 (33.5)	20 (10.8)	8 (5,13)
**DSM IV—Dependence question 3: During the times when you drank alcohol, did you end up drinking more than you planned when started?**	Yes	121 (59.9)	73 (60.3)	22 (18.1)	11 (7,17)
	No	81 (40.1)	6 (7.4)	5 (3.7)	5 (3,9)
**DSM IV—Dependence question 4: Have you tried to reduce or stop drinking alcohol but failed?**	Yes	55 (27.2)	47 (85.5)	11 (20.0)	12 (7,19)
	No	147 (72.8)	32 (21.8)	14 (9.5)	7 (5,12)
**DSM IV—Dependence question 5: On the days that you drank, did you spend substantial time in obtaining alcohol, drinking, or in recovering from the effects of alcohol?**	Yes	33 (16.3)	33 (100)	6 (18.2)	16 (9,19)
	No	169 (83.7)	46 (27.2)	19 (11.2)	7 (5,12)
**DSM IV—Dependence question 6: Did you spend less time working, enjoying hobbies or being with others because of your drinking?**	Yes	36 (17.8)	34 (94.4)	6 (16.7)	13 (8,19)
	No	166 (82.2)	45 (27.1)	19 (11.5)	7 (5,12)
**DSM IV—Dependence question 7: If your drinking caused you health or mental problems, did you still keep on drinking?**	Yes	13 (6.4)	10 (76.9)	4 (30.8)	12 (11,18)
	No	189 (93.6)	69 (36.5)	21 (11.1)	8 (5,13)

**Table 3:** Sensitivity and specificity of MINI results compared with PEth results.

Populations	Dependence by DSM IV		PEth-16:0/18:1 ≥0.30 µmol/L		Area under Receiver Operating Characteristics (AUROC)
			Positive	Negative	
**Overall (*n* = 202)**	≥3 dependence questions	Yes	15 (60.0) ^1^	64 (36.2)	0.62 (0.52–0.72)
		No	10 (40.0)	113 (63.8) ^2^	
**Female college students (*n* = 41)**	≥3 dependence questions	Yes	0 (0.0)	6 (15.4)	0.42 (0.37–0.48)
		No	2 (100.0)	33 (84.6)	
**Male college students (*n* = 58)**	≥3 dependence questions	Yes	4 (57.1)	16 (31.4)	0.63 (0.42–0.84)
		No	3 (42.9)	35 (68.6)	
**Male casual labourers (*n* = 103)**	≥3 dependence questions	Yes	11 (68.8)	42 (48.3)	0.60 (0.47–0.73)
		No	5 (31.3)	45 (51.7)	
**Overall (*n* = 202)**	≥4 dependence questions	Yes	10 (40.0)	29 (16.4)	0.62 (0.52–0.72)
		No	15 (60.0)	148 (83.6)	
**Female college students (*n* = 41)**	≥4 dependence questions	Yes	0 (0.0)	3 (7.7)	0.46 (0.42–0.50)
		No	2 (100.0)	36 (92.3)	
**Male college students (*n* = 58)**	≥4 dependence questions	Yes	3 (42.9)	4 (7.8)	0.68 (0.47–0.88)
		No	4 (57.1)	47 (92.2)	
**Male casual labourers (*n* = 103)**	≥4 dependence questions	Yes	7 (43.8)	22 (25.3)	0.59 (0.46–0.73)
		No	9 (56.2)	65 (74.7)	
**Overall (*n* = 202)**	≥5 dependence questions	Yes	3 (12.0)	9 (5.1)	0.54 (0.47–0.60)
		No	22 (88.0)	168 (94.9)	
**Female college students (*n* = 41)**	≥5 dependence questions	Yes	0 (0.0)	1 (2.6)	0.49 (0.46–0.51)
		No	2 (100.0)	38 (97.4)	
**Male college students (*n* = 58)**	≥5 dependence questions	Yes	1 (14.3)	1 (2.0)	0.56 (0.42–0.70)
		No	6 (85.7)	50 (98.0)	
**Male casual labourers (*n* = 103)**	≥5 dependence questions	Yes	2 (12.5)	7 (8.0)	0.52 (0.43–0.61)
		No	14 (87.5)	80 (92.0)	

^1^ Sensitivity, ^2^ Specificity.

Given that the MINI is frequently applied as a diagnostic tool for alcohol dependence, high specificity is a desired test feature. However, the low sensitivity is a matter of concern as a PEth-16:0/18:1 level of ≥0.30 µmol/L clearly suggests recent heavy chronic alcohol intake [26].

In consequence, in order to address harmful drinking among young people in East Africa and elsewhere, the AUDIT questionnaire remains a sensitive tool to identify problematic levels of alcohol use, and AUDIT scores of 8 or more should trigger efforts towards targeted health education [28]. Attempting to diagnose alcohol dependence using the original version of the MINI can however not be recommended due to its lack of specificity; more stringent cut-offs or a modified version of the MINI are required. However, the sensitivity of any of these options is low and health workers need to be aware that the application might be associated with a false negative diagnosis.

The new version of diagnostic statistical manual the DSM-5 [6] still includes the two questions that pose specificity problems in the diagnosis of dependence among young people in general [29], and as shown this applies also to our part of east Africa. The concerns we described for the MINI/DSM IV would remain when the DSM-5 would be applied to young people.

**Table 4:** Sensitivity and specificity of modified MINI results compared with PEth results.

Populations	Dependence by Modified DSM IV		PEth-16:0/18:1 ≥0.30 µmol/L		Area under Receiver Operating Characteristics (AUROC)
			Positive	Negative	
**Overall (*n* = 202)**	≥1 dependence questions	Yes	16 (64.0) ^1^	76 (42.9)	0.61 (0.50–0.71)
		No	9 (36.0)	101 (57.1) ^2^	
**Female college students (*n* = 41)**	≥1 dependence questions	Yes	0 (0.0)	8 (20.5)	0.40 (0.33–0.46)
		No	2 (100.0)	31 (79.5)	
**Male college students (*n* = 58)**	≥1 dependence questions	Yes	4 (57.1)	20 (39.2)	0.59 (0.38–0.80)
		No	3 (42.9)	31 (60.8)	
**Male casual labourers (*n* = 103)**	≥1 dependence questions	Yes	12 (75.0)	48 (55.2)	0.60 (0.48–0.72)
		No	4 (25.0)	39 (44.8)	
**Overall (*n* = 202)**	≥2 dependence questions	Yes	10 (40.0)	35 (19.8)	0.60 (0.50–0.70)
		No	15 (60.0)	142 (80.2)	
**Female college students (*n* = 41)**	≥2 dependence questions	Yes	0 (0.0)	3 (7.7)	0.46 (0.42–0.50)
		No	2 (100.0)	36 (92.3)	
**Male college students (*n* = 58)**	≥2 dependence questions	Yes	3 (42.9)	6 (11.8)	0.66 (0.45–0.86)
		No	4 (57.1)	45 (88.2)	
**Male casual labourers (*n* = 103)**	≥2 dependence questions	Yes	7 (43.8)	26 (29.9)	0.57 (0.44–0.70)
		No	9 (56.2)	61 (70.1)	
**Overall (*n* = 202)**	≥3 dependence questions	Yes	3 (12.0)	9 (5.1)	0.54 (0.47–0.60)
		No	22 (88.0)	168 (94.9)	
**Female college students (*n* = 41)**	≥3 dependence questions	Yes	0 (0.0)	1 (2.6)	0.49 (0.46–0.51)
		No	2 (100.0)	38 (97.4)	
**Male college students (*n* = 58)**	≥3 dependence questions	Yes	1 (14.3)	1 (2.0)	0.56 (0.42–0.70)
		No	6 (85.7)	50 (98.0)	
**Male casual labourers (*n* = 103)**	≥3 dependence questions	Yes	2 (12.5)	7 (8.0)	0.52 (0.43–0.61)
		No	14 (87.5)	80 (92.0)	

^1^ Sensitivity, ^2^ Specificity.

Our study has some limitations. Firstly, there might be inter-individual differences not always making it possible to link PEth concentration to a precise drinking level and this may imply a potential misclassification of subjects, leading to an under- or over-diagnosis of problem drinking [16]. Secondly, not all cases of chronic heavy alcohol use may reflect alcohol dependence; *vice versa*, there may be genuinely alcohol dependent individuals who have not used alcohol for some weeks and will therefore become PEth negative. Thirdly, the PEth cut-off level applied in our study is the one for heavy alcohol use established for the Swedish population [21]. Fourthly, responses to questions on reported alcohol use may be subject to social desirability bias. Drinking behaviour may have been underreported, but we can also not exclude that the amount of alcohol taken may have been exaggerated by some young people, e.g., out of a desire to impress interviewers. To minimize these types of bias, we ensured that interviewers and interviewees were of the same gender, that study staff were comparatively young themselves, and were trained to provide a friendly and conducive atmosphere during the interview. The variation of sensitivity and specificity between gender groups could be associated with the limitations of PEth described above but also with possible genuine differences between groups in their response to screening questions as observed with other alcohol screening tools [30].

Given the high but still increasing problem of harmful alcohol use among young people in low-income settings [1], the already pressing need for interventions at population and individual level will become even more urgent in future. An accurate tool for the diagnosis of alcohol dependence is needed both for epidemiological and clinical purposes, and it will be crucial in facilitating appropriate care for alcohol dependent young people. Our study shows that the MINI is not an adequate tool to detect alcohol dependence among young people in Africa.

## 5. Conclusions

Using the specific and sensitive alcohol biomarker PEth as reference for heavy drinking, the performance of the MINI in detecting alcohol dependence among young people from northern-Tanzania is was unsatisfactory. Specificity was improved using a modified version but sensitivity remained low. An accurate tool for the diagnosis of alcohol dependence is needed for epidemiological and clinical purposes.

## Section I2. Alcohol Dependence

**In the past 12 months:**

**Table:**

**a**	Did you need to drink more in order to get the same effect that you got when you first started drinking?	**Yes/No**
**b**	When you cut down on drinking did your hands shake, did you sweat or feel agitated? Did you drink to avoid these symptoms or to avoid being hungover, for example, "the shakes", sweating or agitation? If **Yes** to either, CODE **Yes**.	**Yes/No**
**c**	During the times when you drank alcohol, did you end up drinking more than you planned when you started?	**Yes/No**
**d**	Have you tried to reduce or stop drinking alcohol but failed?	**Yes/No**
**e**	On the days that you drank, did you spend substantial time in obtaining alcohol, drinking, or in recovering from the effects of alcohol?	**Yes/No**
**f**	Did you spend less time working, enjoying hobbies, or being with others because of your drinking?	**Yes/No**
**g**	Have you continued to drink even though you knew that the drinking caused you health or mental problems?	**Yes/No**

Are 3 or more I2 answers CODED Yes? If Yes: *Current* *alcohol dependence.* Reproduced with permission of Dr. David Sheehan [31].
